# Heat stress reduces growth rate of red deer calf: Climate warming implications

**DOI:** 10.1371/journal.pone.0233809

**Published:** 2020-06-01

**Authors:** F. J. Pérez-Barbería, A. J. García, J. Cappelli, T. Landete-Castillejos, M. P. Serrano, L. Gallego

**Affiliations:** Game and Livestock Resources Unit, University of Castilla-La Mancha, IDR, IREC, Albacete, Spain; Universidad de la Republica Uruguay, URUGUAY

## Abstract

Climate models agree in predicting scenarios of global warming. In endothermic species heat stress takes place when they are upper their thermal neutral zone. Any physiological or behavioural mechanism to mitigate heat stress is at the cost of diverting energy from other physiological functions, with negative repercussions for individual fitness. Tolerance to heat stress differs between species, age classes and sexes, those with the highest metabolic rates being the most sensitive to stressing thermal environments. This is especially important during the first months of life, when most growth takes place. Red deer (*Cervus elaphus*) is supposedly well adapted to a wide range of thermal environments, based on its worldwide distribution range, but little is known about the direct effect that heat stress may have on calf growth. We assessed the effect that heat stress, measured by heat stress indices and physical environment variables (air temperature, relative air humidity, wind speed and solar radiation), have on calf and mother body weights from calf´s birth to weaning. We used 9265 longitudinal weekly body weight records of calf and mother across 19 years in captive Iberian red deer. We hypothesised that (i) heat stress in hot environments has a negative effect on calf growth, especially in males, as they are more energetically demanding to produce than females; and that (ii) the body weight of the mother through lactation should be negatively affected by heat stress. Our results supported hypothesis (i) but not so clearly hypothesis (ii). By weaning (day 143) calves growing under low heat stress environment grew up to 1.2 kg heavier than those growing in high heat stress environment, and males were more affected by heat stress than females. The results have implications in animal welfare, geographical clines in body size and adaptation to climate change.

## Introduction

Climate projections from computer models indicate that by 2100 average global temperature is highly likely to rise between 1 and 3.7°C [1]. Under this scenario, ruminants are expected to be affected, directly and indirectly, because of changes in quantity and quality of food, water availability, agro-ecological zones, increase in heat stress and diseases [2]. Simultaneously, ruminants impact on climate change, because of their enteric methane emissions [3, 4], which account for about 25% of methane emissions worldwide [1]. Heat stress on ruminants has received a great deal of attention, probably because it is the immediate animal response to changes in its thermal environment. There is a range of ambient temperatures within which an animal experiences no regulatory changes in metabolic heat production or evaporative heat loss, and it is known as thermo-neutral zone TNZ [5, 6]. TNZ condition implies an animal´s energy expenditure is at its minimum, for a specific behavioural activity and physiological state, and it varies across species and individuals. Heat stress takes place when an animal is in a physical environment above its TNZ [7, 8]. An animal responds to heat stress with adaptive mechanisms, some of them are fast responses, such as behavioural (changes in body position, activity budget, foraging), physiological (respiratory rate, core temperature, heart beating, sweating), neuro-endocrine, blood biochemical, metabolic, cellular and molecular; while other responses require long-term changes, such as morphological adaptations (size, coat insulation, changes in allometric size relations between body parts) [9]. When an animal is exposed to temperatures upper its lethal thermal maximum, even for short periods of time, adaptive mechanisms are no longer effective and the animal dies [10]. Any adaptive mechanism an animal uses to acclimatise to the new thermal environment implies energy costs [11]. If the cost is such that it can be offset using body reserves, it will not affect animal´s fitness [9], otherwise, food nutrient utilization, intake, animal production, reproduction, health and mortality will be affected by heat stress [2].

Red deer is the most widely distributed deer species in the world. Its natural range covers Europe, south western Asia and North Africa, and it has been successfully extended via introduction of populations to Australia, New Zealand and Argentina [12]. Red deer live under a wide range of physical environment conditions, from cold and windy exposure in mountain ranges, to hot and dry in Mediterranean habitats. Despite its good adaptation to a variety of climatic conditions [13], deer stresses under critical combinations of wind speed, solar radiation, air temperature and humidity [14]. There are numerous studies that have assessed the performance of ungulate populations in response to climatic variables, especially those associated with climate change [15, 16]. Less effort has been dedicated to assess short term effects of heat on individual traits in wild species, because of the logistical problems involved in collecting this data [17, 18, 8].

Red deer is an economically important species in Europe [19, 20], as it provides a sustainable resource of meat and income via hunting and eco-tourism to economically depressed and depopulated rural areas [21]. Spain has probably the largest red deer population in Europe [19, 4], the higher numbers are concentrated in the west between 38–40° latitude, where maximum temperatures during the first few months of a calf´s life can reach 45°C (www.aemet.es).

In highly polygynous species, such as the red deer, males are larger than females [22, 23]. This sexual dimorphism starts from birth and increases rapidly during the first months of life [24, 25]. Calves of the sex more expensive to produce, ie. males, are more sensitive to limitations in energy supply than female calves [26, 27, 28], so it is expected that under heat stress conditions males´ growth suffered more than females´ growth. Lactation in red deer hinds is energetically demanding, it can double the energy requirements of advance pregnancy [29], which might made body condition of lactating hinds more susceptible to heat stress than body condition of dry hinds.

Using longitudinal data of body weight of mother and offspring during lactation in a captive population of Iberian red deer, together with physical environment variables and heat stress indices, we aimed to assess if heat stress in a hot environment affects calves´ growth and mothers´ condition across lactation. The predictions are: (1) heat stress has a negative effect on calf growth rate, stronger on males, as they are energetically more expensive to produce than females; (2) mother´s body condition, expressed as body weight, decreases through lactation due to the high energy demands of producing milk, but especially under heat stress ambient conditions.

## Methods

### Study area and animals

Data collection was carried out at the University of Castilla-La Mancha (UCLM) deer farm experimental facilities (38°57'32.8"N 1°52'51.8"W, Albacete, Spain,) between 2000 and 2018. The UCLM deer farm is a scientific facility orientated to the study of life history traits, reproduction, nutrition and antler growth in Iberian red deer. The study used 583 mother-offspring pairs (Table 1), comprising 150 different mothers. Mothers age ranged between 1 and 17 years (mean = 5.9, sd = 3.64).

**Table 1:** Number of mother-calf red deer pairs across the study.

year	2000	2001	2002	2003	2004	2005	2006	2007	2008	2009	2010	2011	2012	2013	2014	2015	2016	2017	2018
n	10	7	7	6	35	35	51	65	71†	52	41†	39	38	15	15	20	23	21	28†

† one mother bearing twins.

Mother-calf pairs were split into 2–3 groups and each was allocated to different fields of approx. 0.5 ha. The animals relied entirely on supplementary feed, as the amount of grass provided by the fields was negligible. The base diet year-round was a well-balanced mixture of chopped alfalfa hay and orange pulp supplied *ad libitum* three times a week, and between March and October was supplemented with pelleted feed. Feed was presented to deer on both-side access 14 m long belt feeders to minimise aggressions during feeding. Animals had free access to water at all times. Each plot was fenced with a stock-proofed wire 2 m height that supported a corrugated metal sheet 1.2 m height. Shade was provided by the projection created by the corrugated metal sheet, two roofed bin-feeder sheds in each plot (51 m^2^) and a line of trees around one third of the perimeter of each plot. This meant that shade was available all day long but it was reduced to approx. 200 m^2^ in each plot at solar noon during the experimental period. Water sprinklers were available during summer. The farm complied with Spanish animal welfare legislation. It was daily attended by qualified personnel and an expert deer veterinarian looked after the animals on a weekly basis.

### Age and body weight

Body weight of calves and mothers was monitored between 10 and 22 times (mean = 17.7, sd = 3.08, S1 Fig) through a period of 143 days between births and forced weaning. Monitoring of body weight took place weekly (mean = 8.2 day, sd = 3.52, range = 1–22, S2 Fig), comprising a total of 9265 body weights across 486 monitoring events.

At each monitoring event animals were gathered in the fields and driven to the handling facility through a system of alleys, where they were weighed on a plate platform scale fitted with a motion hold sensor. Calves at birth were weighed in the field, in close presence of their mothers to minimise stress, using a digital portable scale (all weights ± 0.01 kg). The procedure was supervised by a veterinarian specialised on deer. Body weight monitoring was part of the farming activities to control the condition and welfare of the animals. It was a procedure under no experimental license requirements. The animal work carried out in this study was approved by the Animal Welfare and Ethical Review Body of the scientific establishment (Comité de Ética en Experimentación Animal CEEA, UCLM).

### Heat stress indices and physical environment data

The most reliable method of assessing heat stress is by calorimetry techniques. Calorimetry measures the heat produced from metabolic activity, using direct or indirect methods [30]. The most extensively used indirect methods are those based on respirometry, which estimates metabolic activity by determining rates of O_2_ consumption and/or CO_2_ production of animals caged in respiration chambers. Respirometry techniques enable measuring changes in metabolic rate related to animal physiology, intake, diet composition, and experimental changes in the respiration chamber physical environment [31]. To overcome the logistic problems of using calorimetry techniques to simulate outdoor physical environment and assessing how they affect body condition, reproduction and animal production [4, 32], many indices and proxies of thermal stress that have been developed. Some proxies of thermal stress rely on changes in physiological behaviour, such as breath frequency and panting behaviour [33, 34, 35]. Other methods use indirect measurements of the energy expenditure and heat flux necessary to maintain homeothermy, based on operative temperature mechanical models [7, 8]. Among the thermal stress indices widely used are those that combine air temperature, air humidity and wind speed, on the basis that homeothermic species offset the exposure to high temperature by sweating, as when water evaporation occurs on the skin surface, the energy removed from the vaporized water reduces body temperature. Evaporation is highly reduced when atmospheric water vapour is close to saturation, and increases with wind, as it facilitates the removal of water vapour from the air layer surrounding the skin, preventing the creation of insulating epiclimatic conditions on the skin surface [5].

We used meteorological data from the Spanish Ministry of Agriculture, Food and Environment, supplied by SIAR regional service of Castilla-La Mancha (available at http://crea.uclm.es/siar/datmeteo/). Data came from the meteorological station of Albacete (38°56'56.5"N 1°53'53.3"W), only 2 km from the UCLM experimental deer farm and located at the same altitude. We used daily mean records across the study period of air temperature, relative humidity and wind speed, and global solar radiation accumulated per day (Eqs 1 and 2, below).

There are many indices that attempt to express heat stress experienced by animals [5]. The most commonly used, with satisfactory results in a variety of conditions, is the temperature-humidity index (THI, Eq 1) that combines temperature and humidity [36, 37]. Among some of THI variants there is one (THIWS, Eq 2) that incorporates the convection effect of wind speed, and solar radiation [38, 36], which might be worth considering in outdoors conditions,

THI=0.8×T+0.01×Hr×(T−14.4)+46.4 Eq 1

THIWS=4.51+THI−(1.992×W)+1.887×SR Eq 2

where T is the daily mean temperature (°C), Hr is the mean relative humidity (%), W is the mean wind speed (m∙s^-1^) and SR is the accumulated solar radiation across a 24 h circadian period (MJ∙m^-2^).

### Statistical analysis

We used two different statistical approaches, (i) non-linear mixed regression models to assess the effects of heat stress on calf body weight [39], and (ii) generalised additive mixed linear models GAM on mother weight [40]. This was justified because it is well accepted that calf growth fits an exponential asymptotic curve (Eq 3) [24, 41, 25], which can be efficiently parameterised using non-linear mixed regression models implemented with the R software package nlme [39]. On the other hand, there is not a defined model that represents the change in mother body weight through lactation. Consequently, we applied GAM regression models that are flexible on the shape of the response. GAM models were implemented using the R software package mgcv with multiple smoothing parameter estimation by restricted marginal likelihood [42].

Both analyses used two types of explanatory terms, animal and physical environment variables. The animal terms were the calf age (days) and mother age (years), calf sex and their pertinent interactions, and were fitted as fixed effects in GAM and non-linear mixed effects models. In addition, calf and mother identity, and calf cohort were fitted as random effects. The second type of variables fitted in the above models were those that represent heat environment. More concretely, the two indices of heat stress defined above (THI, THIWS), and the aforementioned four physical environment variables (T, Hr, W, SR). Although heat stress indices are widely used in the literature [43], as any other composite index, they have the problem that when used in statistical analyses the particular relationships and error structures between the response variable and the variables that constitute the index are lost [44]. For the analysis of calf weight we undertook three non-linear mixed effects models fitting (i) animal variables and THI; (ii) animal variables and THIWS; and (iii) animal and physical environment variables. Similarly, for mother weight but using GAM models.

Although GAM models are additive by definition, we fitted interactions between explanatory variables using the ti function within GAM model formulae. This produces a tensor product interaction which is appropriate when the main effects and any lower interactions are also present [45, 46]. To deal with independent random effects, these were treated as smooths (using “re” basis in model formulae), the terms produce a parametric interaction of the predictors and penalize the corresponding coefficients with a multiple of the identity matrix, corresponding to an assumption of normality [45]. We assumed that the response could be non-linear but relatively simple, and because penalized regression smoothers gain computational efficiency using a basis of modest size [45] we used small k values (k = 3, 4). The non-linear mixed models evaluated an asymptotic regression function and its gradient of the type,

$$f(x)=Asym+(R0-Asym)\times e^{{-e}^{lrc}\times t}$$ Eq 3

where t is calf age in days; Asym represents the horizontal asymptote; R0 is the response at t = 0; and lrc is the natural logarithm of the rate constant. The starting values of the parameters of this function were initially estimated using calves body weights against age through lactation using the self-starting model implemented in the nonlinear least-squares regression package (nlm) of R software [47]. The starting parameters of the other explanatory variables and their pertinent interactions were guessed and fitted in nlme, the new estimated parameters by the model were fitted as starting parameters and the model was re-run to corroborate consistency in the parametrisation of the model, which was achieved in all cases. Model selection was performed using Akaike (AIC) weights aided by the normalised probability of the Kullback–Leibler discrepancy ratio; in which model A is to be preferred over competing model B [48]. The coefficients of the linear mixed model were calculated using REML, as the estimates are more accurate than using maximum likelihood [49]. To improve the readability of the models output we plotted the response of interest by fixing the other explanatory variables to their mean values. Graphics were constructed in R [47] using the ggplot2 package based on The Grammar of Graphics [50].

## Results

### Physical environment

**Fig 1:**
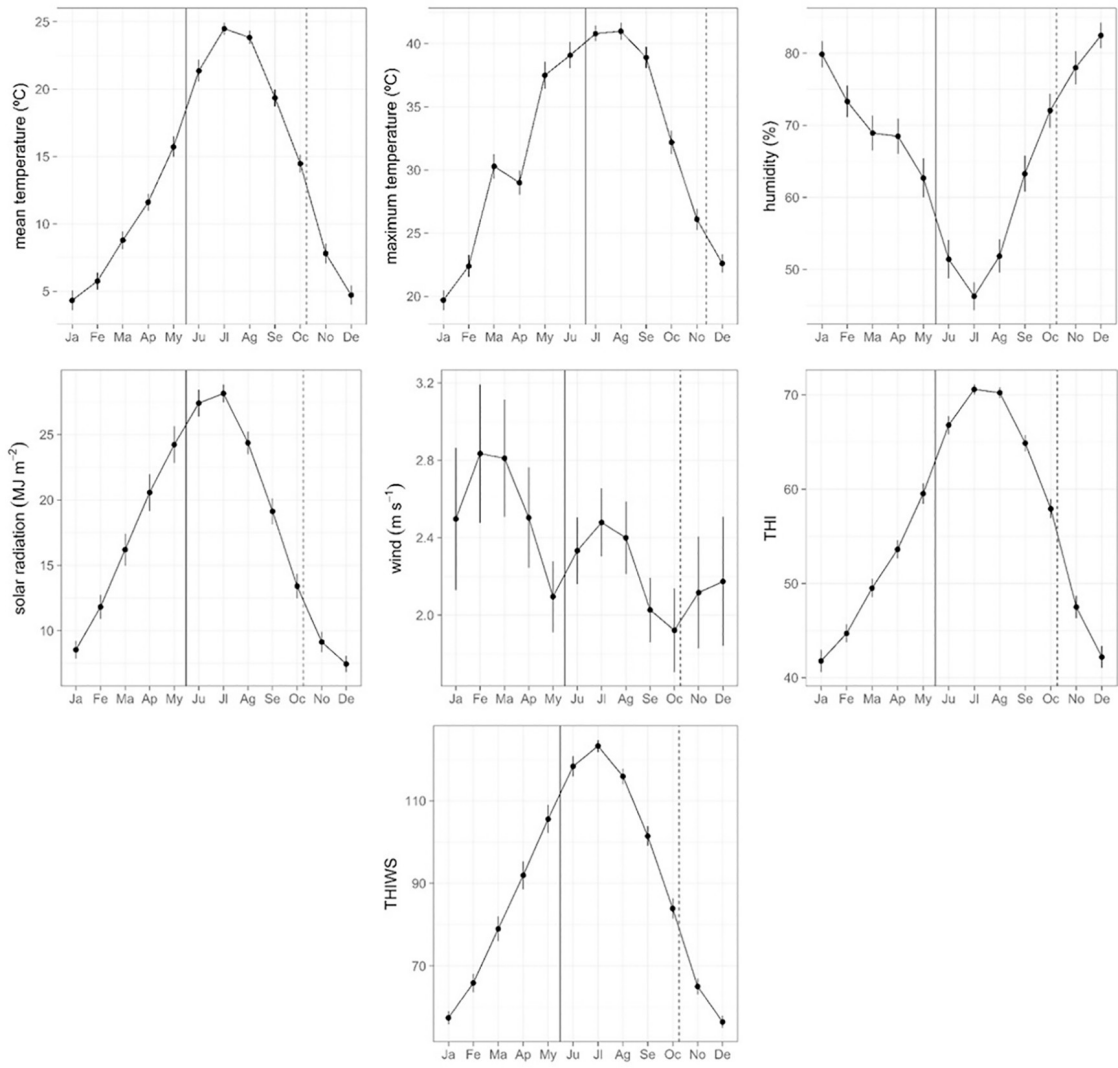
From left to right top to bottom: Monthly mean and standard errors of mean and maximum daily air temperature, relative air humidity, accumulated daily solar radiation, mean wind speed and two indices of heat stress (THI, THIWS) in the study area (period 1999–2018).

The mean date of birth was 23th of May (sd = 10.2 d, n = 583) and through the next 143 days till weaning and across the 19 years of the study, mothers and offspring experienced the hottest, driest and highest solar radiation days year round (mean temperature = 24.5°C in July; maximum temperature = 41.0°C in August; air humidity = 46.2% in July; solar radiation = 28.1 MJ∙m^-2^ in July, Fig 1). This period comprised average annual wind speed values (2.35 m∙s^-1^) and also the lowest wind speed records year round (minimum = 1.92 m∙s^-1^, Fig 1). The highest records in thermal stress took place between June and September, as indicated by THI index (maximum = 123.3 in July), and through June and August as indicated by THIWS index (maximum = 70.6 in July) (Fig 1).

### Heat stress on calf growth

**Fig 2:**
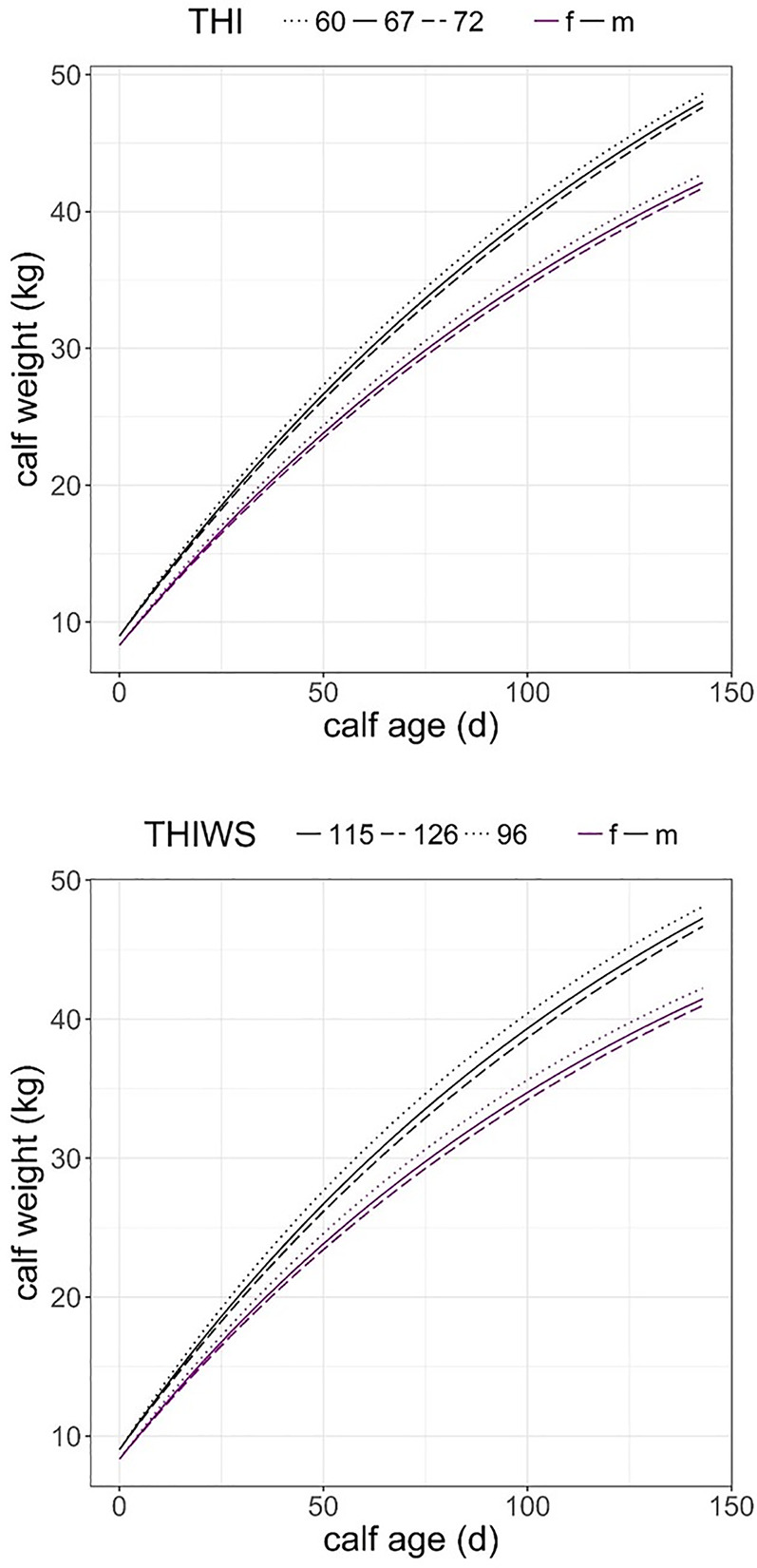
Predicted calf growth from birth to forced weaning (day 143) from models in Tables 2 and 3 for three values of THI heat stress index (60, 67, 72) corresponding to its quantiles 0.1, 0.5 and 0.9; and three values of THIWS (96, 115, 126) corresponding to quantiles 0.1, 0.5 and 0.9; f: female calf (magenta); m: male calf (black).

Our final model clearly indicates that THI index had a negative effect on the rate of calf growth (estimate = -0.009, se = 0.001, p < 0.001, Table 2, Fig 2) and affected males and females in the same fashion (lrc x sex [male] estimate = -0.002, se = 0.001, p = 0.173, Table 2). For females of average body weight (99 kg) and age (6 yr) the model predicted birth weights of 8.3 kg and 9.0 kg for females and males, respectively. At weaning time the predicted calf body masses for THI = 67 (0.5 quantile) were 42.1 kg for females and 48.0 kg for males. Calves of both sexes were 0.6 kg heavier than average calf weight when they experienced low heat stress (THI = 60, 0.1 quantile) but were 0.4 kg lighter than average calf weight under high heat stress (THI = 72, 0.9 quantile) (Table 2, Fig 2). In relation to the animal variables fitted in the same model, the growth rate of males was higher than that in females (estimate [male] = 0.008, se = 0.002, p = 0.001), and calf growth rate increased with mother age following AIC information criterion but not corroborated by p-values (estimate = 0.117, se = 0.135, p = 0.391). After controlling for the effect of mother age, mother weight had a negative effect of the rate of calf growth (estimate = - 0.001, se = 0.0001, p = 0.023), and as calf grew older its rate of growth decreased, only confirmed by AIC criterion (estimate = - 0.001, se = 0.0001, p = 0.137).

**Table 2:** Coefficients and statistics of an exponential asymptotic mixed linear model on calf weight (Kg) controlling for animal and a thermal stress index (THI) variables.

Random effects	model 0	model 1	model 2	model 3	model 4	final model				
Groups (sd)										
Asym intercept calf cohort	5.68	5.73	6.17	6.26	5.41	5.40				
Asym intercept mother	8.06	6.69	6.73	6.68	5.88	5.93				
Asym intercept calf	9.76	9.31	9.35	9.23	8.07	8.04				
Residual	1.44	1.43	1.42	1.42	1.41	1.41				
Fixed effects						estimate	se	df	t-value	*P*
Asym intercept	Y ***	Y ***	Y ***	Y ***	Y ***	50.433	9.716	8659	5.19	<0.001
Asym mother wt	Y ***	Y ***	Y ***	Y ***	Y ***	0.299	0.030	8659	9.97	<0.001
Asym sex (male)	—	Y ***	Y ***	Y ***	Y ***	-0.443	5.146	8659	-0.08	0.931
Asym mother age (yr)	—	—	Y ***	Y ***	Y ***	-0.724	0.150	8659	-4.80	<0.001
Asym calf age (d)	—	—	—	Y	Y **	-0.169	0.056	8659	-2.96	0.003
Asym THI	—	—	—	—	Y ***	0.186	0.059	8659	3.16	0.002
Asym THI x sex (male)	—	—	—	—	—	0.179	0.076	8659	2.35	0.019
R0 intercept	Y ***	Y ***	Y ***	Y ***	Y ***	6.129	0.621	8659	9.85	<0.001
R0 mother wt	Y ***	Y ***	Y ***	Y ***	Y ***	0.025	0.002	8659	8.43	<0.001
R0 sex (male)	—	Y ***	Y ***	Y ***	Y ***	0.683	0.078	8659	8.68	<0.001
R0 mother age (yr)	—	—	Y ***	Y ***	Y ***	0.059	0.010	8659	5.67	<0.001
R0 calf age (d)	—	—	—	Y ***	Y	-0.004	0.003	8659	-1.30	0.192
lrc intercept	Y ***	Y ***	Y ***	Y ***	Y ***	-4.100	0.175	8659	-23.36	<0.001
lrc mother wt	Y **	Y **	Y **	Y ***	Y *	-0.001	0.0001	8659	-2.26	0.023
lrc sex (male)	—	Y *	Y *	Y *	Y	0.117	0.136	8659	0.85	0.391
lrc mother age (yr)	—	—	Y ***	Y ***	Y ***	0.008	0.002	8659	3.90	0.001
lrc calf age (d)	—	—	—	Y *	Y	-0.001	0.0001	8659	-1.48	0.137
lrc THI	—	—	—	—	Y ***	-0.009	0.001	8659	-6.29	<0.001
lrc THI x sex (male)	—	—	—	—	—	-0.002	0.001	8659	-1.36	0.173
AIC	35966.4	35733.7	35583.5	35514.4	35408.4	35405.1				
ΔAIC	561.3	328.5	178.4	109.2	3.32	0				
weights AIC	1.08E-122	3.82E-72	1.52E-39	1.59E-24	0.16	0.84				
Kullback–Leibler	1	1	1	1	0.84	—				

Asym: horizontal asymptote; R0: response at calf age = 0; lrc: natural logarithm of the rate constant. THI: thermal stress index based on air temperature and air humidity; Y: term included in the model, Model 0–4 and final: six competing models in the model selection process; calf age: in days; mother age: in years; calf cohort: calf year of birth; df: degrees of freedom; AIC: Akaike information criterion; wAIC: AIC weights of the model; ΔAIC: delta AIC; Kullback–Leibler discrepancy ratio: normalised probability that reference model in column *i* is to be preferred over model in column *i + 1*; significance of the terms p-value: *** = 0–0.001, ** = 0.001–0.01, * = 0.01–0.05, + = 0.05–0.1. Female is the sex level of reference.

Similar results were obtained when using THIWS heat stress index (Table 3, Fig 2), except that THIWS had a stronger negative effect on the calf growth rate of males than on females, as indicated by the significant interaction lrc × calf sex (estimate [male] = - 0.001, se = 0.001, p = 0.041). Model predictions on birth weight for females and males were 8.3 kg and 9.0 kg, respectively. Predicted calf body masses at weaning for THIWS = 115 (0.5 quantile) were 41.5 kg (female) and 47.3 kg (male), females were 0.7 kg and males 0.8 kg heavier when they were in an environment of low heat stress THIWS = 96 (0.1 quantile). Contrasting with 0.5 kg and 0.6 kg lighter, females and males respectively, under a high heat stress environment (THIWS = 126, 0.9 quantile) (Table 3, Fig 2). Calf growth rate responded to animal variables in the same fashion that THI index did, that is, male growth rate was higher than female growth rate, calf growth rate increased with mother age, and decreased with mother weight and with calf age (Table 3).

**Table 3:** Coefficients and statistics of an exponential asymptotic mixed linear model on calf weight (Kg) controlling for animal and a thermal stress index (THIWS) variables. THIWS: stress index based on air temperature, humidity, wind speed and solar radiation; Female is the sex level of reference. Acronyms as in Table 2.

Random effects	model 0	model 1	model 2	model 3	model 4	final model				
Groups (sd)										
Asym intercept calf cohort	5.68	5.73	6.17	6.26	4.98	4.98				
Asym intercept mother	8.06	6.69	6.73	6.68	5.40	5.43				
Asym intercept calf	9.76	9.31	9.35	9.23	7.42	7.41				
Residual	1.44	1.43	1.42	1.42	1.41	1.41				
Fixed effects						Estimate	se	df	t-value	*P*
Asym intercept	Y ***	Y ***	Y ***	Y ***	Y ***	51.127	7.990	8659	6.398	<0.001
Asym mother wt	Y ***	Y ***	Y ***	Y ***	Y ***	0.254	0.025	8659	10.091	<0.001
Asym sex (male)	—	Y ***	Y ***	Y ***	Y ***	2.090	2.834	8659	0.737	0.461
Asym mother age (yr)	—	—	Y ***	Y ***	Y ***	-0.564	0.131	8659	-4.279	<0.001
Asym calf age (d)	—	—	—	Y	Y **	-0.148	0.049	8659	-3.006	0.003
Asym THIWS	—	—	—	—	Y ***	0.076	0.018	8659	4.038	0.001
Asym THIWS x sex (male)	—	—	—	—	—	0.072	0.024	8659	2.984	0.003
R0 intercept	Y ***	Y ***	Y ***	Y ***	Y ***	5.979	0.626	8659	9.539	<0.001
R0 mother wt	Y ***	Y ***	Y ***	Y ***	Y ***	0.025	0.003	8659	8.570	<0.001
R0 sex (male)	—	Y ***	Y ***	Y ***	Y ***	0.690	0.076	8659	9.031	<0.001
R0 mother age (yr)	—	—	Y ***	Y ***	Y ***	0.058	0.010	8659	5.609	<0.001
R0 calf age (d)	—	—	—	Y ***	Y	-0.003	0.003	8659	-1.061	0.288
lrc intercept	Y ***	Y ***	Y ***	Y ***	Y ***	-4.041	0.145	8659	-27.697	<0.001
lrc mother wt	Y **	Y **	Y **	Y ***	Y +	-0.001	0.000	8659	-1.914	0.056
lrc sex (male)	—	Y *	Y *	Y *	Y	0.122	0.097	8659	1.256	0.209
lrc mother age (yr)	—	—	Y ***	Y ***	Y ***	0.006	0.002	8659	3.420	0.001
lrc calf age (d)	—	—	—	Y *	Y *	-0.002	0.001	8659	-2.631	0.009
lrc THIWS	—	—	—	—	Y ***	-0.004	0.001	8659	-8.058	<0.001
lrc THIWS x sex (male)	—	—	—	—	—	-0.001	0.001	8659	-2.047	0.041
AIC	35966.43	35733.66	35583.53	35514.35	35421.7	35417.32				
ΔAIC	549.11	316.34	166.21	97.03	4.38	0				
weights AIC	5.18E-120	1.83E-69	7.26E-37	7.63E-22	0.10	0.90				
Kullback–Leibler	1	1	1	1	0.90	—				

**Fig 3:**
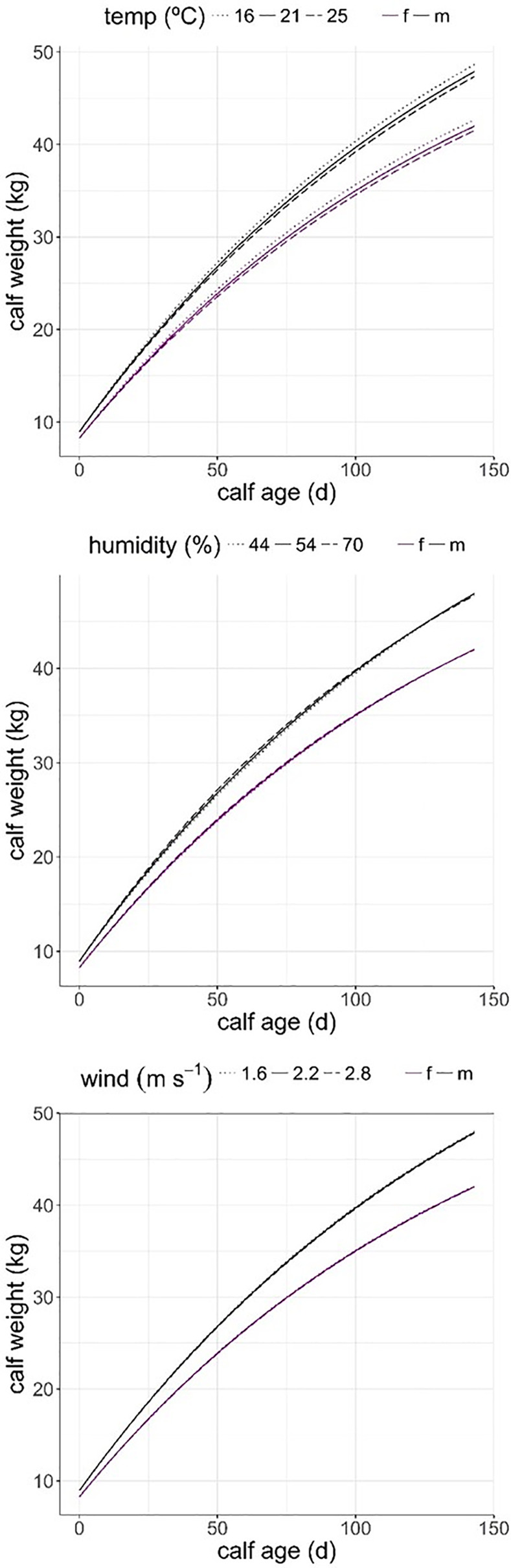
Predicted calf growth from birth to forced weaning (day 143) from model in Table 4, for three values of mean daily air temperature (16°C, 21°C, 25°C) corresponding to quantiles 0.1, 0.5 and 0.9; air humidity (quantiles 0.1 = 44%; 0.5 = 54%; 0.9 = 70%) and wind speed (quantiles 0.1 = 1.6 m∙s^-1^; 0.5 = 2.2 m∙s^-1^ and 0.9 = 2.8 m∙s^-1^); f: female calf (magenta); m: male calf (black).

Table 4 shows the contribution of animal variables (mother and calf age, mother weight and calf sex) together with physical environment variables (T, Hr, SR, W) to calf growth. The final model included all animal variables and physical environment variables except SR and the interactions SR x calf sex and W x calf sex on AIC model selection basis (Table 4). High temperature and strong wind hampered calf growth, as indicated by the negative estimates of these two coefficients in the model (T estimate = - 0.006, se = 0.003; p = 0.054; W estimate = - 0.02, se = 0.001, p = 0.069), while air humidity had a positive effect on calf growth rate (Hr estimate = 0.003, se = 0.001, p = 0.009, Fig 3). Air temperature had the stronger effect on calf growth rate of all physical environment variables, as shown in Fig 3. Calf growth rate responded to animal variables in a similar way to that described for THI and THIWS stress indices models (Table 4). S3 Fig shows actual data of males and females growth against calf age, highlighting the large variability across records. Calf growth increased asymptotically with calf age and this effect was hampered as air temperature increased, but less in males than in females as indicated by the interaction T × calf sex selected on AIC basis, though not supported by p-value significance (male calf effect estimate = 0.002; se = 0.004; p = 0.609; Table 4, Fig 3). Air Humidity had the inverse effect, the more humid the environment the faster calf growth was (estimate = 0.003; se = 0.001; p = 0.009; Fig 3), this effect was stronger in males than in females (male estimate: 0.003; se = 0.001; p = 0.034; Table 4). Wind speed had a small additive negative effect on male growth, though this was not corroborated by p-value significance (male estimate: -0.02; se = 0.001; p = 0.070, Fig 3).

**Table 4:** Coefficients and statistics of an exponential asymptotic mixed linear model on calf weight (kg) controlling for animal and climatic variables. Female is the sex level of reference. Acronyms as in Table 2.

Random effects	model 0	model 1	model 2	model 3	model 4	model 5	model 6	model 7	final model				
Groups (sd)													
Asym intercept calf cohort	5.33	5.33	5.28	5.27	4.93	4.94	4.93	4.92	5.24				
Asym intercept mother	5.79	5.84	5.71	5.73	5.39	5.38	5.37	5.39	5.72				
Asym intercept calf	7.94	7.92	7.78	7.78	7.33	7.33	7.32	7.31	7.76				
Residual	1.41	1.41	1.40	1.40	1.40	1.40	1.40	1.40	1.40				
Fixed effects									estimate	se	df	t-value	*P*
Asym intercept	Y ***	Y ***	Y ***	Y ***	Y ***	Y ***	Y ***	Y ***	66.68	9.44	8653	7.06	0
Asym mother wt	Y ***	Y ***	Y ***	Y ***	Y ***	Y ***	Y ***	Y ***	0.26	0.02	8653	9.81	0
Asym sex (male)	Y ***	Y *	Y *	Y ***	Y ***	Y *	Y *	Y	20.29	6.26	8653	3.24	0.0012
Asym mother age (yr)	Y ***	Y ***	Y ***	Y ***	Y ***	Y ***	Y ***	Y ***	-0.67	0.13	8653	-4.79	0
Asym calf age (d)	Y **	Y **	Y **	Y **	Y ***	Y ***	Y ***	Y ***	-0.15	0.05	8653	-2.92	0.0035
Asym T	Y ***	Y ***	Y	Y	Y	Y	Y	Y	0.006	0.11	8653	0.05	0.9595
Asym Hr	—	—	Y ***	Y *	Y	Y	Y	Y	-0.09	0.04	8653	-2.33	0.0194
Asym SR	—	—	—	—	Y **	Y	Y	Y	—	—	—	—	—
Asym W	—	—	—	—	—	—	Y	Y +	0.41	0.39	8653	1.05	0.2931
Asym T x sex (male)	—	Y **	Y *	Y	Y	Y	Y	Y	-0.06	0.15	8653	-0.40	0.6841
Asym Hr x sex (male)	—	—	—	Y **	Y **	Y +	Y +	Y	-0.14	0.05	8653	-2.58	0.0098
Asym SR x sex (male)	—	—	—	—	—	Y	Y	Y	—	—	—	—	—
Asym W x sex (male)	—	—	—	—	—	—	—	Y ***	—	—	—	—	—
R0 intercept	Y ***	Y ***	Y ***	Y ***	Y ***	Y ***	Y ***	Y ***	5.78	0.62	8653	9.26	0
R0 mother wt	Y ***	Y ***	Y ***	Y ***	Y ***	Y ***	Y ***	Y ***	0.02	0.003	8653	8.60	0
R0 sex (male)	Y ***	Y ***	Y ***	Y ***	Y ***	Y ***	Y ***	Y ***	0.68	0.07	8653	8.75	0
R0 mother age (yr)	Y ***	Y ***	Y ***	Y ***	Y ***	Y ***	Y ***	Y ***	0.05	0.01	8653	5.37	0
R0 calf age (d)	Y	Y	Y	Y	Y	Y	Y	Y	-0.003	0.004	8653	-0.75	0.4475
lrc intercept	Y ***	Y ***	Y ***	Y ***	Y ***	Y ***	Y ***	Y ***	-4.69	0.17	8653	-26.71	0
lrc mother wt	Y *	Y *	Y +	Y +	Y +	Y +	Y +	Y	-0.001	0.001	8653	-1.82	0.0675
lrc sex (male)	Y	Y	Y	Y +	Y +	Y	Y	Y	-0.25	0.15	8653	-1.64	0.1009
lrc mother age (yr)	Y ***	Y ***	Y ***	Y ***	Y ***	Y ***	Y ***	Y ***	0.009	0.002	8653	4.37	0
lrc calf age (d)	Y +	Y +	Y *	Y *	Y *	Y *	Y *	Y *	-0.002	0.001	8653	-2.08	0.0374
lrc T	Y ***	Y ***	Y	Y *	Y	Y	Y	Y	-0.006	0.003	8653	-1.92	0.0544
lrc Hr	—	—	Y ***	Y *	Y	Y	Y	Y	0.003	0.001	8653	2.61	0.0089
lrc SR	—	—	—	—	Y ***	Y **	Y *	Y *	—	—	—	—	—
lrc W	—	—	—	—	—	—	Y	Y	-0.02	0.001	8653	-1.81	0.0697
lrc T x sex (male)	—	Y +	Y +	Y	Y	Y	Y	Y	0.002	0.004	8653	0.51	0.6085
lrc Hr x sex (male)	—	—	—	Y *	Y *	Y	Y	Y	0.003	0.001	8653	2.12	0.034
lrc SR x sex (male)	—	—	—	—	—	Y	Y	Y	—	—	—	—	—
lrc W x sex (male)	—	—	—	—	—	—	—	Y *	—	—	—	—	—
AIC	35380.4	35375.7	35353.9	35350.2	35364.8	35367.3	35364.0	35355.0	35346.4				
ΔAIC	33.9	29.3	7.5	3.8	18.4	20.9	17.6	8.6	0				
weights AIC	3.52E-08	3.64E-07	1.94E-02	1.22E-01	8.33E-05	2.42E-05	1.25E-04	0.01	0.85				
Kullback–Leibler	0.91	0.99	0.86	0.00	0.22	0.83	0.98	0.98	—				

### Heat stress on mother weight

**Fig 4:**
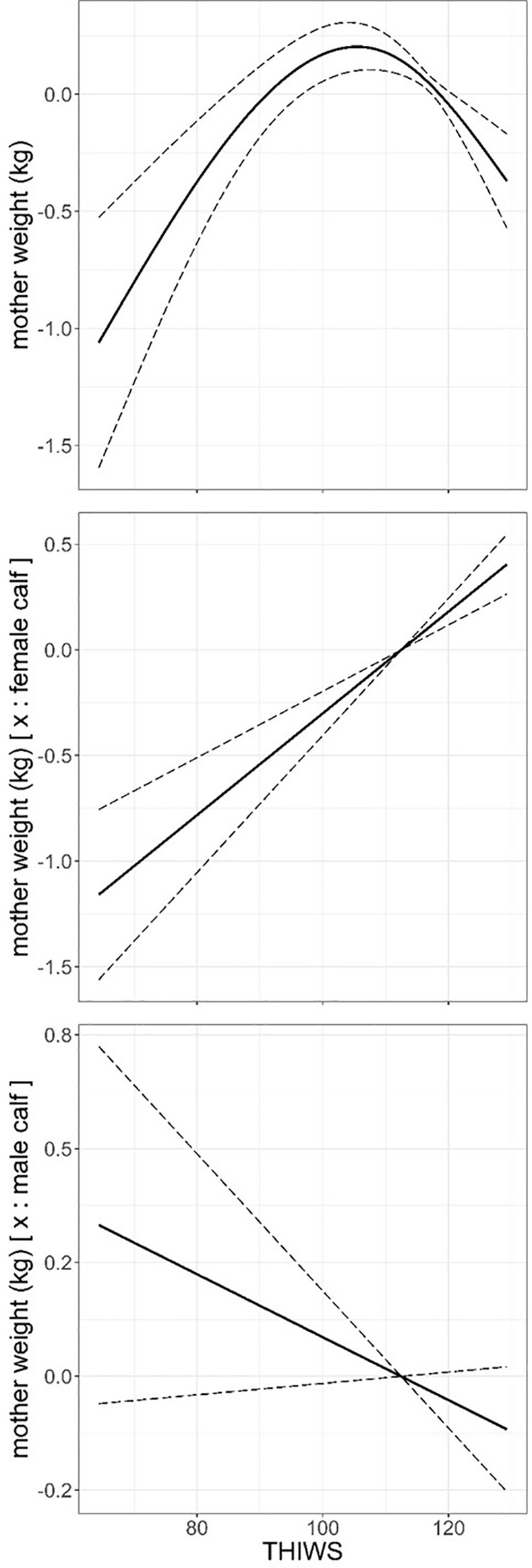
Predicted mother weight (kg) against THIWS thermal stress index and the additive effect of bearing a male or a female through lactation from model in Table 5. Dashed line: ± standard error. Response variable has been centred by subtraction of the mean value.

A GAM model that included animal variables and THIWS thermal stress index, revealed that mother weight increased across age (p = 0.043) but it was hampered when mothers bore a male in comparison with those that bore a female. The latter only supported by AIC model selection criterion but not by p-values (Table 5, S4 Fig). Mothers lost an average of 0.4 kg of body weight through lactation (p < 0.001), this effect was stronger when mothers bore males than when bearing females (p <0.001, Table 5, S4 Fig). Mother weight responded in a complex fashion to THIWS (p < 0.001), namely, mother weight increased on an average of 1.2 kg within 65–107 THIWS range, and above 107 up to 130 mother weight decreased by an average of 0.5 kg (Table 5, Fig 4). THIWS had a detrimental additive effect on the weight of the mothers that bore a male as compared with those bearing a female (Fig 4). A GAM model that replaced THIWS with THI produced consistent results with those described above, therefore THI model is not shown.

**Table 5:** Parametric, smooth coefficients and statistics of GAM models on red deer mother weight (Kg) together with animal and a thermal stress variable (THIWS).

	model 0	model 1	model 2	model 3	model 4	final model				
k	3	3	3	3	3	3				
Parametric coefficients	terms in the model					estimate	se	t-value	p	
intercept	Y ***	Y ***	Y ***	Y ***	Y ***	95.1285	4.3714	21.761	<0.0001	
sex (male)	Y	Y	Y	Y	Y	-0.4852	1.2077	-0.402	0.688	
Smooth terms						coefficients of the smooths †				
						coeff.1	coeff.2	df	F	p
calf age (d)	Y ***	Y ***	Y ***	Y ***	Y ***	0.77	-2.35	1.67	112.10	<0.001
mother age (yr)	—	Y	Y	Y	Y *	1.21	2.35	0.67	6.13	0.043
THIWS	—	—	—	—	Y ***	1.13	-0.13	1.66	15.11	0.002
calf age x sex (female)	—	—	Y ***	Y ***	Y ***	-0.18	-0.34	0.67	19.41	<0.001
calf age x sex (male)	—	—	Y ***	Y ***	Y ***	-0.62	-1.18	0.67	217.50	<0.001
mother age x sex (female)	—	—	—	Y	Y +	-0.24	4.10	1.66	1.08	0.217
mother age x sex (male)	—	—	—	Y	Y	-0.41	-0.79	0.67	0.50	0.564
THIWS x sex (female)	—	—	—	—	—	1.03	0.67	0.67	49.62	<0.001
THIWS x sex (male)	—	—	—	—	—	-0.30	-0.19	0.67	4.30	0.090
Random effects										
mother	Y ***	Y	Y	Y +	Y			149	1.86E+05	<0.001
calf cohort	Y ***	Y	Y	Y	Y			19	8.07E+05	0.141
calf	Y ***	Y	Y	Y	Y			581	1.07E+04	0.959
deviance explained %	93.4	93.4	93.4	93.4	93.5	93.5				
AIC	48999.7	49003.8	48975.1	48972	48953.6	48946				
ΔAIC	53.6584	57.7608	29.134	25.9606	7.63283	0				
weights AIC	2.18E-12	2.81E-13	4.62E-07	2.26E-06	0.02	0.98				
Kullback–Leibler	0.11	1	0.83	1	0.98	—				

k: number of knots to be used for basis construction

(†) coefficients pertain to the smooth function of the model. Acronyms as in Table 2.

**Fig 5:**
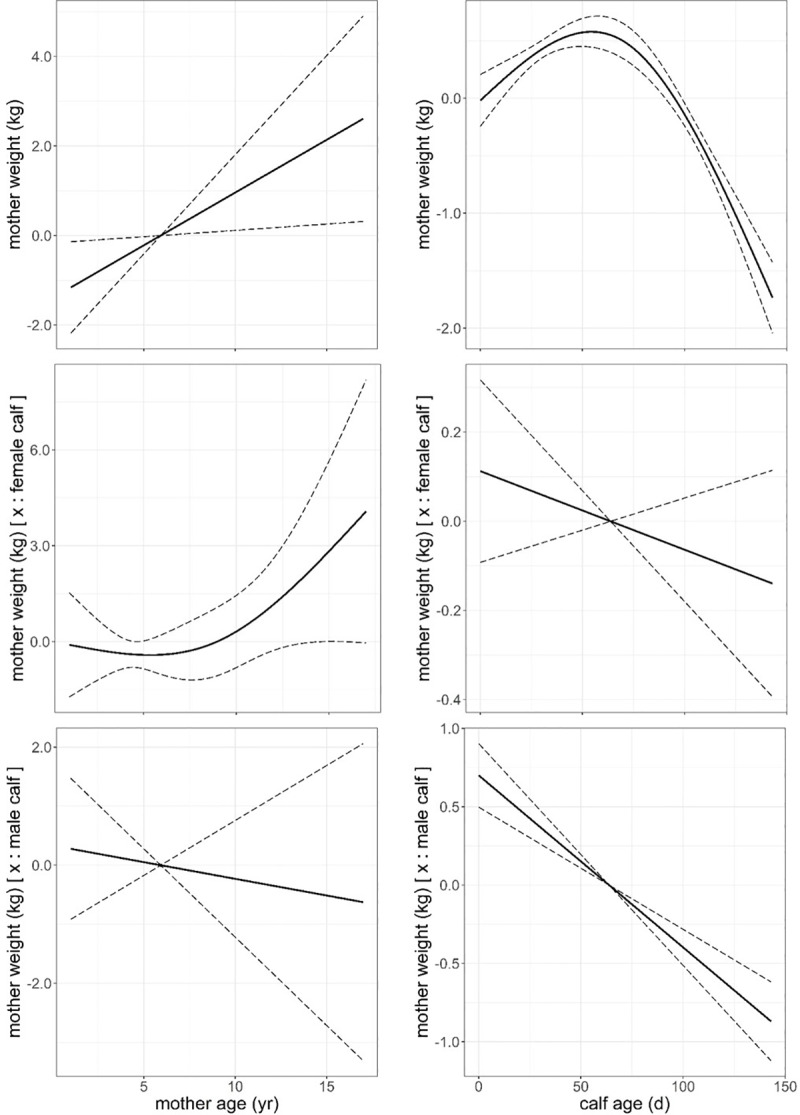
Predicted mother weight (kg) against mother age (years) and calf age (days) and the additive effect of bearing a male or a female through lactation from model in Table 6. Dashed line: ± standard error. Response variable has been centred by subtraction of the mean value.

**Fig 6:**
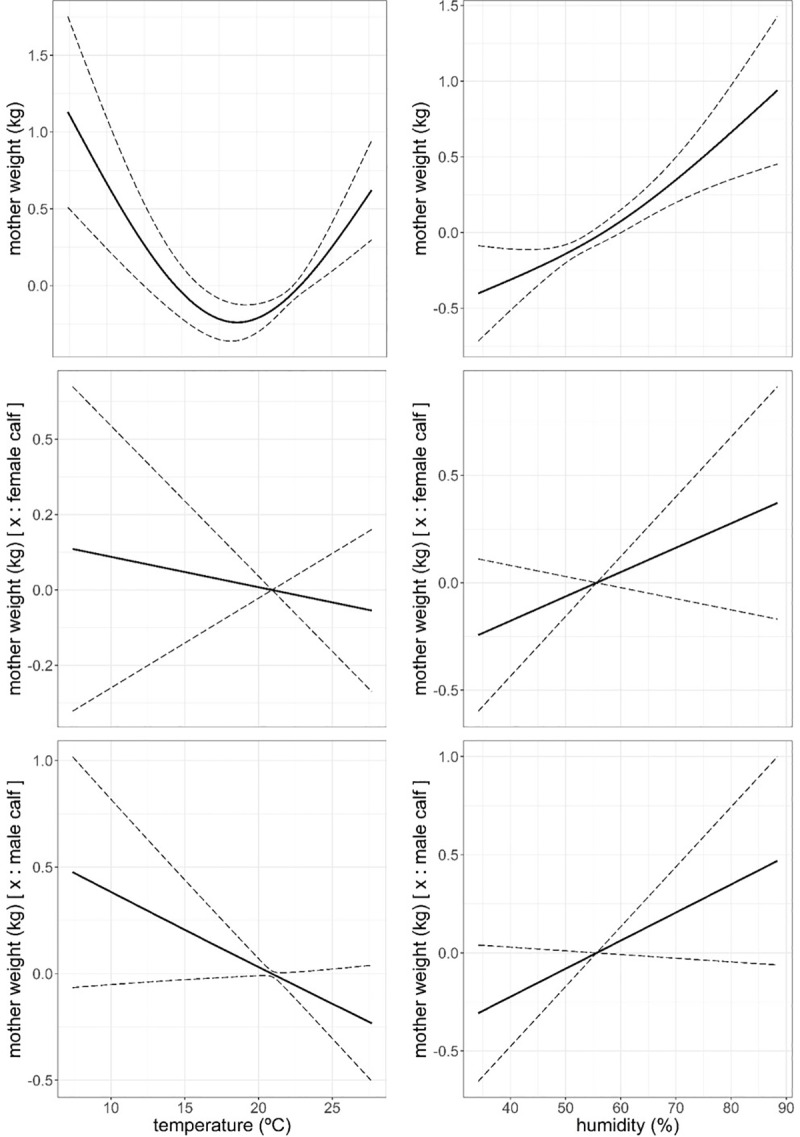
Predicted mother weight (kg) against daily mean air temperature (°C) and percentage of air humidity and the additive effect of bearing a male or a female through lactation from model in Table 6. Dashed line: ± standard error. Response variable has been centred by subtraction of the mean value.

**Fig 7:**
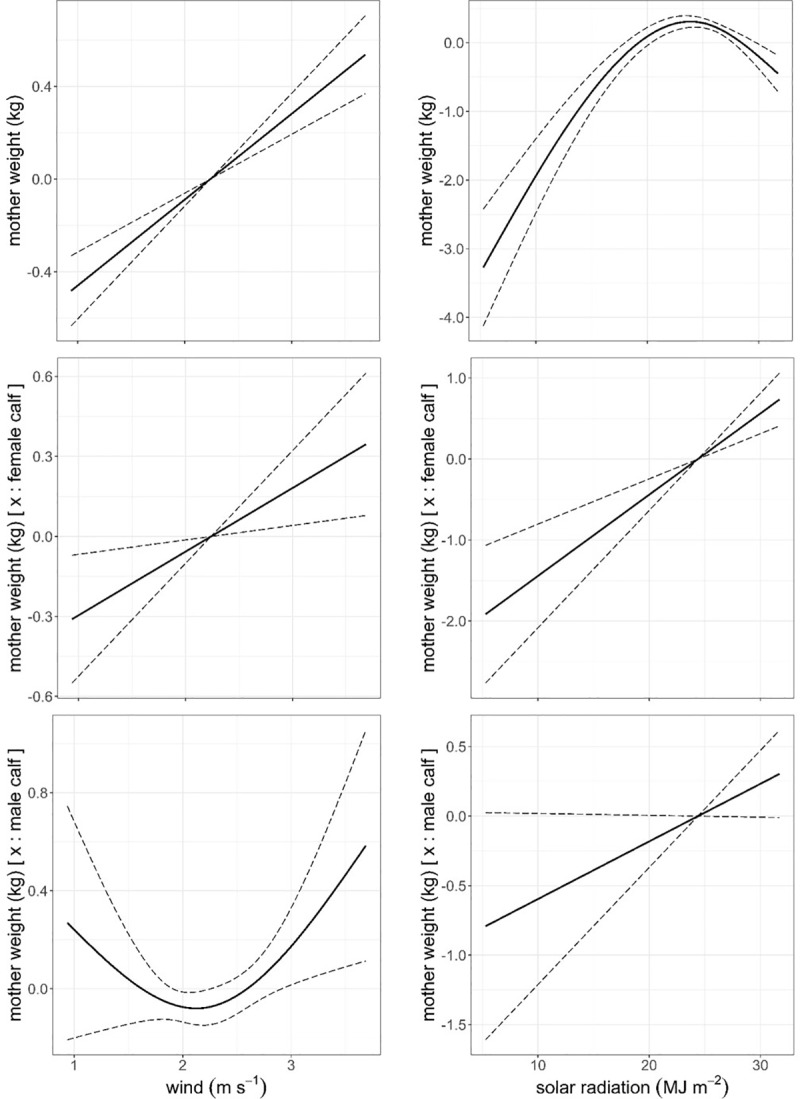
Predicted mother weight (kg) against daily mean wind speed (m∙s^-1^) and solar radiation (MJ∙m^-2^) and the additive effect of bearing a male or a female through lactation from model in Table 6. Dashed line: ± standard error. Response variable has been centred by subtraction of the mean value.

A GAM model that fitted animal and physical environment variables (Table 6) revealed that mothers gained on average 0.6 kg in weight during the first 60 days of lactation, to decrease an average of 2.3 kg by the time of weaning (p < 0.001), bearing a calf had a negative effect on mothers weight, especially when bearing a male (p < 0.001) in comparison with those mothers bearing a female. Mothers weight increased with mother age (p = 0.023) at a lower rate when the mother was bearing a male than a female, with this only corroborated by AIC model selection basis (Fig 5). The model indicated that mother weight responded to physical environment variables in a more complex fashion than the response of calf growth to the same set of physical variables. Mother weight responded in a quadratic fashion to air temperature (p = 0.002), while the response to humidity and wind speed was positive and almost linear (p < 0.001, Figs 6 and 7). Mother weight response to solar radiation followed a similar pattern to that described for THIWS model above, that is, mother weight increased within solar radiation values between 5 and 24 MJ∙m^-2^ and decreased for higher values of solar radiation (p < 0.001, Fig 7). In general, the physical environment variables had a more detrimental effect on mothers that bore a male than on mothers bearing a female (Table 6, Figs 6 and 7).

**Table 6:** Parametric, smooth coefficients and statistics of GAM models on red deer mother weight (kg) together with animal and climatic variables.

	model 0	model 1	model 2	model 3	model 4	model 5	model 6	model 7	model 8	model 9	model 10	final model				
k	3	3	3	3	3	3	3	3	3	3	3	3				
Parametric coefficients	terms in the model											estimate	se	t-value	p	
intercept	Y ***	Y ***	Y ***	Y ***	Y ***	Y ***	Y ***	Y ***	Y ***	Y ***	Y ***	95.2	4.34	22.0	<0.0001	
sex (male)	Y	Y	Y	Y	Y	Y	Y	Y	Y	Y	Y	-0.452	1.164	-0.388	0.698	
Smooth terms												coefficients of the smooths †				
												coeff.1	coeff.2	df	F	p
calf age (d)	Y ***	Y ***	Y ***	Y ***	Y ***	Y ***	Y ***	Y ***	Y ***	Y ***	Y ***	0.492	-1.732	1.66	39.01	<0.001
mother age (yr)	—	Y	Y	Y	Y *	Y *	Y *	Y	Y *	Y *	Y *	1.351	2.616	0.66	7.75	0.023
calf age x sex (female)	—	—	Y ***	Y ***	Y ***	Y ***	Y ***	Y ***	Y ***	Y ***	Y	-0.084	-0.158	0.66	1.81	0.272
calf age x sex (male)	—	—	Y ***	Y ***	Y ***	Y ***	Y ***	Y ***	Y ***	Y ***	Y ***	-0.524	-0.989	0.66	71.62	<0.001
mother age x sex (female)	—	—	—	Y	Y	Y	Y +	Y	Y	Y	Y	0.064	4.075	1.65	1.09	0.199
mother age x sex (male)	—	—	—	Y	Y	Y	Y	Y	Y	Y	Y	-0.323	-0.627	0.66	0.33	0.641
T	—	—	—	—	Y *	Y ***	Y ***	Y ***	Y **	Y *	Y **	-1.290	0.243	1.65	14.78	0.002
Hr	—	—	—	—	—	Y ***	Y ***	Y ***	Y ***	Y ***	Y ***	0.355	0.959	1.28	12.73	<0.001
SR	—	—	—	—	—	—	Y ***	Y ***	Y ***	Y ***	Y ***	3.313	-0.112	1.66	34.44	<0.001
W	—	—	—	—	—	—	—	Y ***	Y ***	Y ***	Y ***	0.428	0.580	0.66	60.90	<0.001
T x sex (female)	—	—	—	—	—	—	—	—	Y +	Y	Y	-0.103	-0.113	0.66	0.39	0.613
T x sex (male)	—	—	—	—	—	—	—	—	Y ***	Y +	Y *	-0.365	-0.392	0.68	4.46	0.080
Hr x sex (female)	—	—	—	—	—	—	—	—	—	Y	Y	0.226	0.383	0.66	2.83	0.169
Hr x sex (male)	—	—	—	—	—	—	—	—	—	Y **	Y +	0.285	0.483	0.66	4.69	0.077
SR x sex (female)	—	—	—	—	—	—	—	—	—	—	Y ***	1.829	0.931	0.66	30.50	<0.001
SR x sex (male)	—	—	—	—	—	—	—	—	—	—	Y +	0.756	0.384	0.66	5.64	0.053
W x sex (female)	—	—	—	—	—	—	—	—	—	—	—	0.275	0.374	0.66	10.05	0.010
W x sex (male)	—	—	—	—	—	—	—	—	—	—	—	-0.326	0.559	1.62	1.73	0.111
Random effects																
mother	Y ***	Y	Y	Y +	Y ***	Y	Y	Y	Y ***	Y ***	Y ***			149	1.48E+05	<0.001
calf cohort	Y ***	Y	Y	Y	Y	Y	Y	Y	Y +	Y +	Y +			19	3.66E+05	0.138
calf	Y ***	Y	Y	Y	Y	Y	Y	Y	Y	Y	Y			581	9.21E+03	0.909
deviance explained %	93.4	93.4	93.4	93.4	93.4	93.4	93.5	93.5	93.5	93.5	93.5	93.5				
AIC	48999.6	49003.7	48975.1	48971.9	48967.3	48945.0	48890.7	48850.0	48842.7	48842.7	48842.4	48841.84				
ΔAIC	157.81	161.91	133.28	130.11	125.50	103.21	48.87	8.19	0.86	0.89	0.60	0				
weights AIC	1.77E-35	2.27E-36	3.74E-30	1.83E-29	1.83E-28	1.27E-23	8.01E-12	0.01	0.21	0.21	0.24	0.33				
Kullback–Leibler	0.11	0.99	0.83	0.90	0.99	1	1	0.97	0.49	0.53	0.57	—				

T: daily mean temperature (°C); Hr: relative air humidity (%); SR: solar radiation (MJ∙m^-2^); W: wind speed (m∙s^-^1). Female is the sex level of reference. Other acronyms as in Tables 2 and 5.

## Discussion

The results of our study clearly indicate that heat stress reduces the rate of growth of red deer calf, and this seems mainly due to the direct effect that physical environment variables have on offspring energy budget across lactation. It is unlikely attributable to the effect that heat stress may have on mother condition. This conclusion is based on the fact that the two heat stress indices (THI, THIWS), mean air temperature and wind speed had all negative effects on calf growth rate, while heat stress indices, air temperature, wind speed and solar radiation had less clear effects on mother weight during lactation as the response was not linear in some variables.

The results suggest that adult hinds had energy and/or behavioural resources to offset the heat stress at which they were exposed during lactation, as compared with calves that were not capable of completely offsetting the energy expense required to maintain homeothermy without diverting energy resources from growth. Despite this, our models indicate that mother weight suffered at heat stress THIWS >107, mean air temperature >16°C and relative humidity <60%, and increased with wind speed.

Physical environment affected calves´growth depending of maternal traits. The growth of offspring males was more negatively affected by heat stress indices (THIWS) and physical environment variables than the growth of offspring females. Older females produced offspring with faster rates of growth, and after the effect of mother age was controlled for, mother weight had a negative effect on the growth rate of calf. Interestingly, THIWS indicated that males offspring were more sensitive to heat stress than females, supporting the prediction that the sex with the highest energy requirements is the one most affected by a demanding physical environment, supporting Hypothesis 1.

### Physical environment and limitations of heat stress indices

Studies on heat stress focus on three types of animal response [51], (i) behavioural: seeking thermal cover, sheltering from wind, or reducing activity [52, 53, 54]; (ii) physiological: among the most common rectal and skin temperature, heart and respiration rates, and metabolic markers based on stress related hormones [33, 55, 56, 57, 58]; and (iii) animal performance: changes in body condition and animal production, such as body weight and milk yield [59, 52, 53, 60]. Translating these animal responses into energy expenditure without relying on calorimetry measurements is not possible [30], as a compromise, proxies of thermal stress using physical environment variables have been developed; though the limitations on the use of indices has been recognised [44], the main problem being that the particular contribution of the variables that constitute the index disappears within the index. Another problem is that a thermal stress index may have solid foundations in expressing a particular thermal environment, but it is unlike to apply to a wide range of physical environments. The use of multivariate analysis overcomes these problems, as it enables identifying the contribution of each variable while taking into account the effects of all other variables on the responses of interest [61]. We applied a multivariate approach, in which all physical environment variables that constituted our thermal stress indices were fitted into the model. This analysis indicated that the contribution of the different variables was not the same on calf growth than on mother weight. Indeed, some variables did not even have any significant contribution, which suggests they were obsolete as constituents of our heat stress indices. Our analysis revealed that daily means of air temperature and wind speed had a negative effect on calf growth, while the effect of humidity was positive. Air temperature and wind speed effects were as predicted, as these variables are positively correlated with convection heat exchange. The positive effect of humidity on calf growth was unexpected, contrasting with heat stress chart predictions [62]. However, mean humidity during the study (end of May–mid-September) was very low (45–55%) when compared with average values of tropical areas (80%), where air humidity is a major contributor to thermal stress in livestock [43, 55, 63] and probably in wild species. It is plausible that increasing humidity up to a 55% threshold might have contributed to decrease thermal stress, for example, by being a surrogate index of rainfall and so promoting sward growth in our experimental arena, a more dense sward reduces reflective solar radiation from the ground as so mitigates heat [5]. Solar radiation did not have any significant contribution in calf growth after air temperature, humidity and wind speed were controlled for. Yamamoto et al [64] found that neither the rate of heat production nor heart rate of Holstein heifers responded to solar radiation in summer, though rectal temperature increased under no shade conditions. In moose (*Alces alces*), a species which is sensitive to suffer heat stress over 14°C, changes in activity rates were associated to air temperature but not to solar radiation in boreal forest, activity increased at night during hot periods [53].

### Behaviour as mitigation mechanism of heat stress

As we commented above changes in behaviour is one of the animal responses to mitigate heat stress. Heat stress can be offset by active using the weather-sheltering environment provided by thermal cover, for example forest cover. Cook et al [52], using an experimental design in which elk were exposed to winter and summer conditions under different forest cover environments, demonstrated that body condition was not affected by reduced wind speed, elevated nocturnal temperature in winter, and shading from solar radiation in summer, but dense forest cover in winter had a negative effect on deer condition, presumably because of reduction in solar radiation flux. Migratory movement is other behavioural mechanisms to mitigate heat stress. In northern populations of red deer, summer and especially winter habitats increase their suitability to deer because of reducing snow cover in climate warming scenarios [65]. Similarly, in Mediterranean environments, it should be expected a reduction in summer habitat suitability because of heat stress increase, under this scenario, altitudinal migration is a plausible strategy of heat stress mitigation. However, for many populations of Iberian red deer this might be a challenge. A large number of Iberian red deer of the southern Spain populations live in fenced hunting estates [66] that precludes long distance and altitudinal movements. Besides, most of these populations are under unsustainable management that relies on food supplementation during mid and late summer, when plants quality and availability is insufficient to maintain high densities of deer [67, 68]. Over-crowded deer populations aimed to hunting eco-tourism have a negative impact on forest renewal, which reduces natural shade availability in these populations. Though no much canopy area is necessary to provide enough shade for a large number of deer, small canopy area concentrates deer on the limited shade spots, increasing aggregation, which promotes diseases [69], and concentrates the impact of herbivory worsening plant regeneration [70]. Reduction in the density of deer in fenced hunting estates could be a solution to achieve sustainable deer populations under climate warming scenarios, but whether this reduction in deer numbers could maintain these hunting estates economically profitable is arguable [71]. Iberian red deer populations from the north of Spain, which almost entirely dwell on open estates and large public grounds, are likely be in the most favourable conditions to cope with climate warming. These deer populations are harvested at sustainable rates and receive no food supplementation year round. Their densities are fitted to the natural resources available, many of them spread across mountain ranges that enable considerable altitudinal seasonal migration, so they can adapt their spatial movement to plant resources availability and heat stress.

Behavioural response to changes in the physical environment can be fast and flexible. Our experimental plots were fitted with water sprinklers during summer, showering under water sprinklers was an unusual behaviour in mothers and offspring, mud bathing was frequently observed in calves but less in mothers. Parker and Robbins [18] described that panting is the primary means of heat dissipation in hot environments in mule deer (*Odocoileus hemionus*) and elk (*Cervus elaphus*), however, this was not a behaviour that we observed in our experimental animals. The most frequent behaviour, to mitigate heat stress observed in mothers and calves in our experiment during the hottest hours of the day, was to reduce activity by lying down under shade. Studies on skin temperature in *Odocoileus hemionus* suggest that preference for shadow beds to avoid direct solar radiation may be instrumental to reduce thermal stress at environmental temperatures that exceeded 25°C [72]. In desert extreme hot summer conditions, Arabian oryx (*Oryx leucoryx*) laid down in shade in the morning, shortly before air temperature exceeded its core body temperature (38.5°C), and remained in shadow beds until evening, when body temperature exceeded air temperature [73]. In the same study it has been suggested that body heat accumulation (temporal higher core body temperature than mean body temperature) could be a mechanism of water economy in desert conditions, as body cooling down by evaporation through the skin and respiratory tract requires a considerable amount of water that might compromise survival [73]. This reveals how behavioural response to heat stress can have complex physiological implications.

### Climate warming implications on deer body size

Climatic models suggest with medium confidence that in the Northern Hemisphere the period 1983–2012 was the warmest of the last 1400 years. It is believe that this was the product of an uptake of energy by the climate system due to a positive total radiative forcing, being its largest driver an increase in atmospheric CO_2_ since 1750 [1]. Under different scenarios climate models predict an increase in land surface temperature of 1–2°C for the period 2046–2065, and between 1 and 3.7°C for the period 2081–2100 [1]. This climatic pattern is projected in the form of warmer and/or fewer cold days and nights, and warmer and/or more frequent hot days and nights. A similar warming pattern has been described for the Iberian peninsula, annual mean temperature has increased in 0.10°C/decade for the period 1850–2005, annual warming was more affected by the increase in autumn and winter temperatures than those in spring and summer, and there were higher rates of change in maximum than in minimum temperatures [74]. Implications of Iberian peninsula warming during this period and that predicted on models at global scale [1], together with results of our study, suggest that body size in red deer might have been decreasing and will continue to do so. This is, however, a very simplistic projection, as climate warming might act on deer body growth in different directions. For example, it has been claimed that increasing atmospheric CO_2_, because of human GHG emissions, promotes photosynthesis and so plant growth, which in turn increases plant biomass for the use of large herbivores. On the other hand, it is argued that plant growth is not constrained by atmospheric CO_2_ availability but nitrogen input from soil [75], which highlight the complexity of assessing how global warming might affect body size in deer.

Our findings have interesting ecological implications on body size geographical clines. Bergmann´s rule establishes that in cold climates large animals benefit by having a lower surface area to volume ratio than smaller animals, because they radiate less heat per unit of mass and so spend less energy to maintain homeothermy, the opposite being true in warmer environments [76]. There is evidence to support this rule in endotherms [77, 78] although there are alternative explanations to this phenomenon [79, 80]. Our results suggest that heat stress in warm environments penalises body growth during lactation. If body growth is not compensated for later in life, this could contribute to explain an intra-specific cline in body mass increase from warm to cold environments.

Our results indicate that under heat stress conditions, red deer male size is more negatively affected than female size, whether this would have long-term adaptive consequences in body-size remains unknown, as we commented above, food and other compensatory factors might affect body size [81]. However, we could hypothesise that if warming conditions hamper male´s growth more than female´s growth, this could have implications in polygynous mating systems, which are mainly based on sexual differences in body size [82]. Among the implications are (i) changes in parental investment between sexes [83], (ii) reduction of sexual differences in mortality age-dependent [66], (iii) reduction in spatial sexual segregation [84], and (iv) increase in trophic niche sexual overlap [85, 86].

### Implications of heat stress on deer farming

Special effort in developing mechanisms to mitigate heat stress has been undertaken in dairy cattle, breeds with high metabolic rate, because heat stress has a negative effect on intake and milk production, suppresses their immune and endocrine system, enhancing disease susceptibility [87]. Cooling systems based on providing shades, improving ventilation, water sprinklers and evaporation enhancers have been demonstrated to be effective in reducing breath rates by -16 up to -38 breaths/min in cows [33, 34, 35], but less clear is their effect on hormonal stress indices of chronic stress, such as triiodothyronine and cortisol [33].

A simplistic way of assessing the energy costs associated to losses in calf body mass due to heat stress, is by estimating the corresponding energy value of the milk a calf needs to produce changes in body growth associated to values of heat stress index between 0.1 and 0.9 quantiles through the first 143 days of lactation (Fig 2), which is 324 MJ, with a mean of 1126 kJ∙d^-1^ (calculated using allometric equation in Robbins et al [88], Energy kcal∙d^-1^ = 226 Wkg^0.837^). This is an important amount of energy to justify the application of measures to minimize heat stress in captive deer. Spiers and collaborators [58] found in dairy cattle that increasing air temperature 10°C above thermoneutral conditions (20°C) increased rectal temperature by 1.5°C, respiration rate from 60 to 90 breath min^-1^, and across 4 days milk yield dropped from 35 kg∙d^-1^ to 26 kg∙d^-1^ and dry matter intake from 24 kg∙d^-1^ to 12 kg∙d^-1^.

Our study indicates that despite the experimental arena offering cooling opportunities they were not enough to completely offset the effects of heat stress in calf growth. The results justify applying management measures to minimise heat stress in red deer, such as providing shades, good ventilation, baths and water sprinklers, as it is recommended in cattle dairy farms [59]. This is especially advisable in deer farms located in hot environments and where animals do not have free access to waterbodies for bathing, nor plenty of tree cover or enough ground that offers deer a variety of physical environments across which thermal stress could be minimised. Actual climatic data and projections of mean maximum temperature into 2100 in Spain (www.aemet.es) suggest that, north-west areas are those that rarely reach heat stress conditions at present and also less chance of reaching those in the future. Under this scenario it seems advisable to locate deer farms in those areas, which has the additional benefit of having some of the best grazing conditions in Spain [89], and so having to rely less on feed supplementation making deer production more sustainable.

It is advisable to deer managers to consider the provision of thermal cover to reduce thermal stress in deer, which also depends on habitat quality and food availability [14]. Moen [90] pointed out that in fawns and does of white-tailed deer (*Odocoileus virginianus*) under the thermal stress conditions of his experiment, the energy balance predicted should not cause a negative balance as long as food supply was sufficient and the deer could seek shelter. Similarly, it has been highlighted in wild red deer the importance of appropriate food supply and the use of behaviour as means to compensate for potential heat stress [91]. This highlights the importance that food energy supply has when assessing the impact of heat stress on the animal’s energy budget and consequently in the animal performance.

## Supporting information

S1 Fig Frequency distribution of monitoring events across 143 days of lactation in the study period (2000–2018); mean: Continuous vertical line; median = dashed vertical line. (JPEG)

S2 Fig Frequency distribution of days between monitoring events across the study (2000–2018); mean: Vertical line. (JPEG)

S3 Fig Supplementary material. Actual calf weight (kg) versus calf age (d) across the first 143 days of life in males and females of Iberian red deer. Grey triangle: male calf; magenta circle: female calf.
(JPEG)

S4 Fig Supplementary material. Response of mother body weight through lactation (from birth to forced weaning day 143) to mother age (years) and calf age (days) of the final model in Table 5, all other variables in the model fixed at their mean values. Dashed line: ± standard error. Response variable has been centred by subtraction of the mean value.
(JPG)
