# Supplementary figures and images for: Heat stress reduces growth rate of red deer calf: Climate warming implications
Source: PLoS One. 2020 Jun 1;15(6):e0233809. doi: 10.1371/journal.pone.0233809 (PMC7263848; doi:10.1371/journal.pone.0233809)

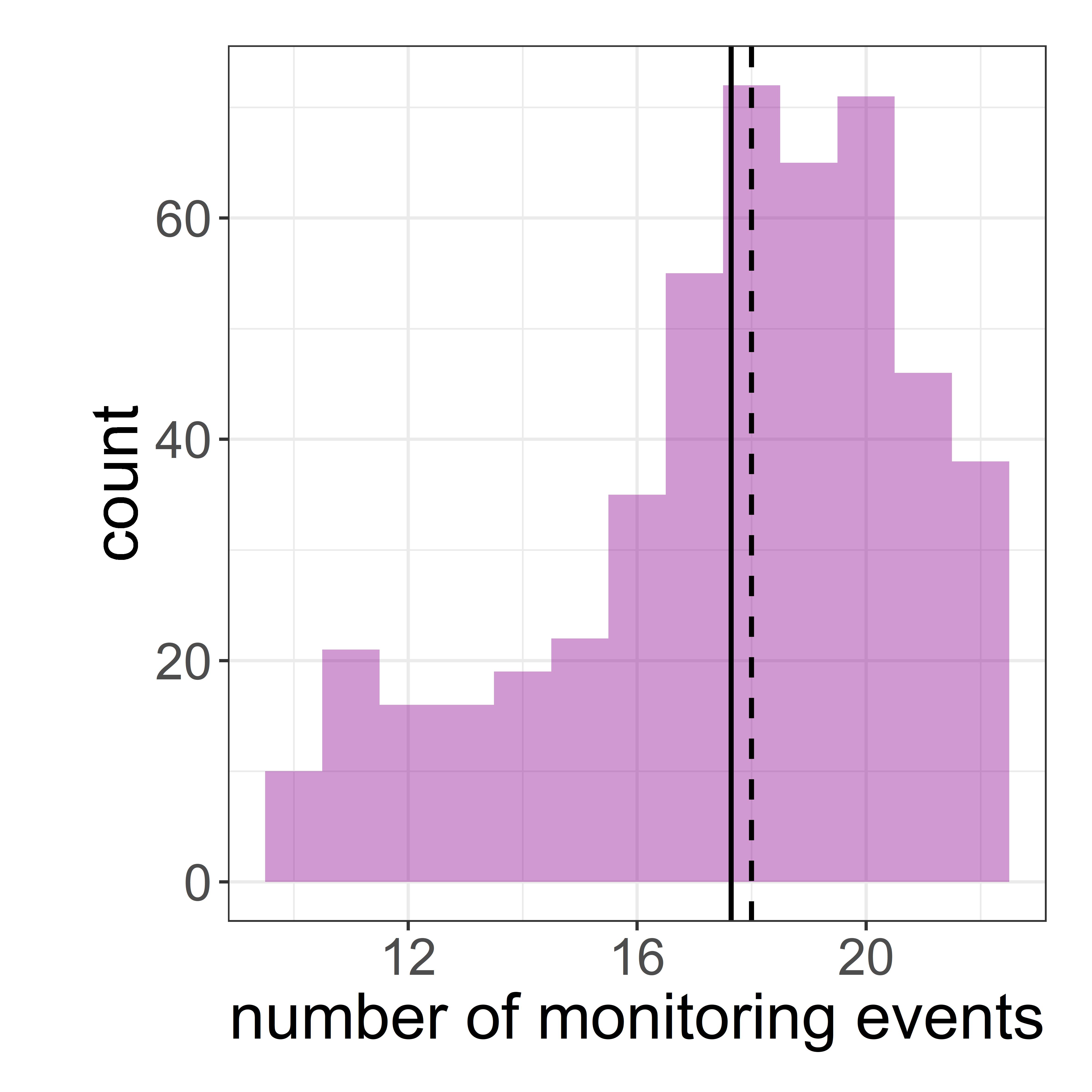

Supplement: S1 Fig — (JPEG) [file pone.0233809.s001.jpeg]

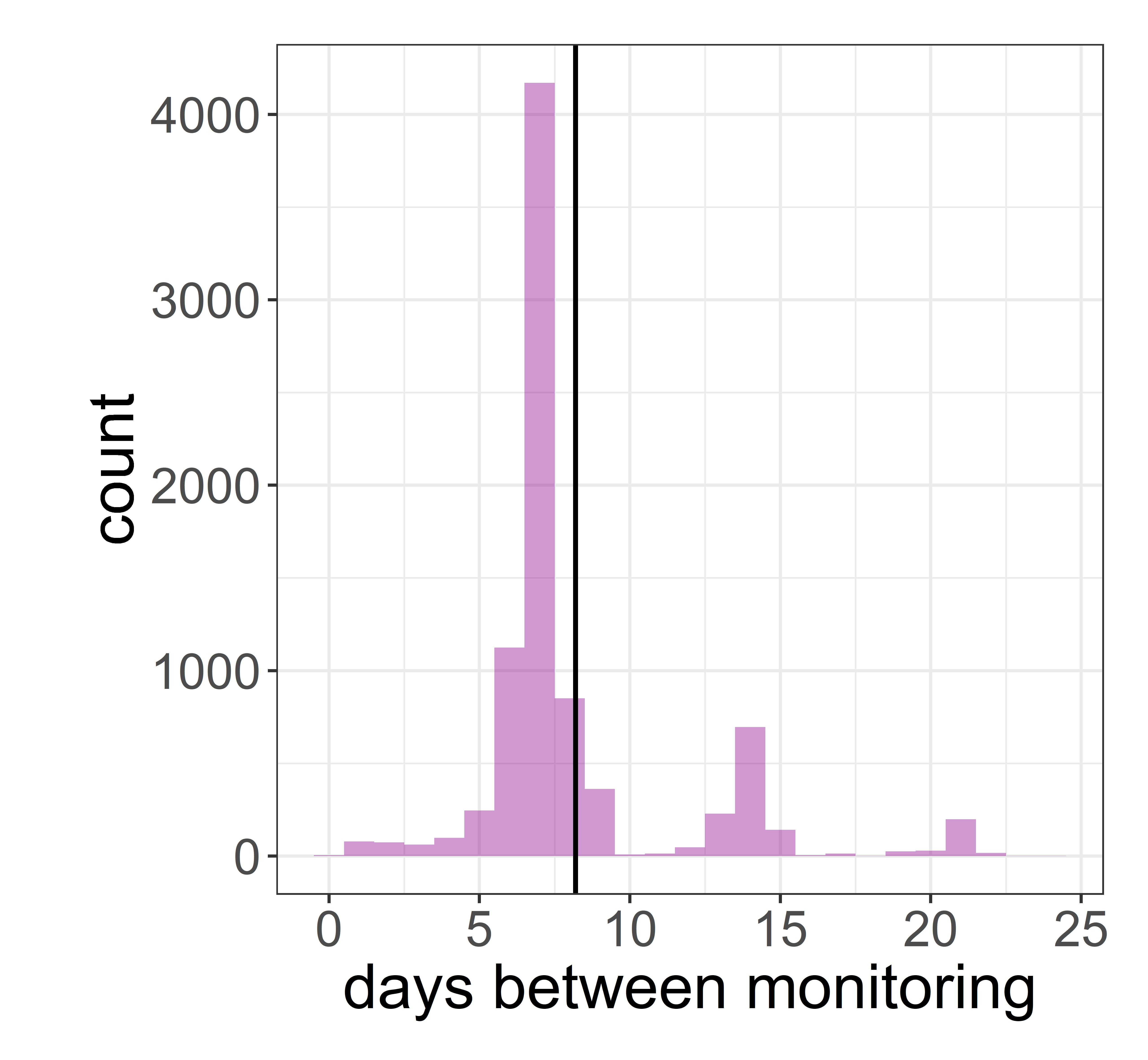

Supplement: S2 Fig — (JPEG) [file pone.0233809.s002.jpeg]

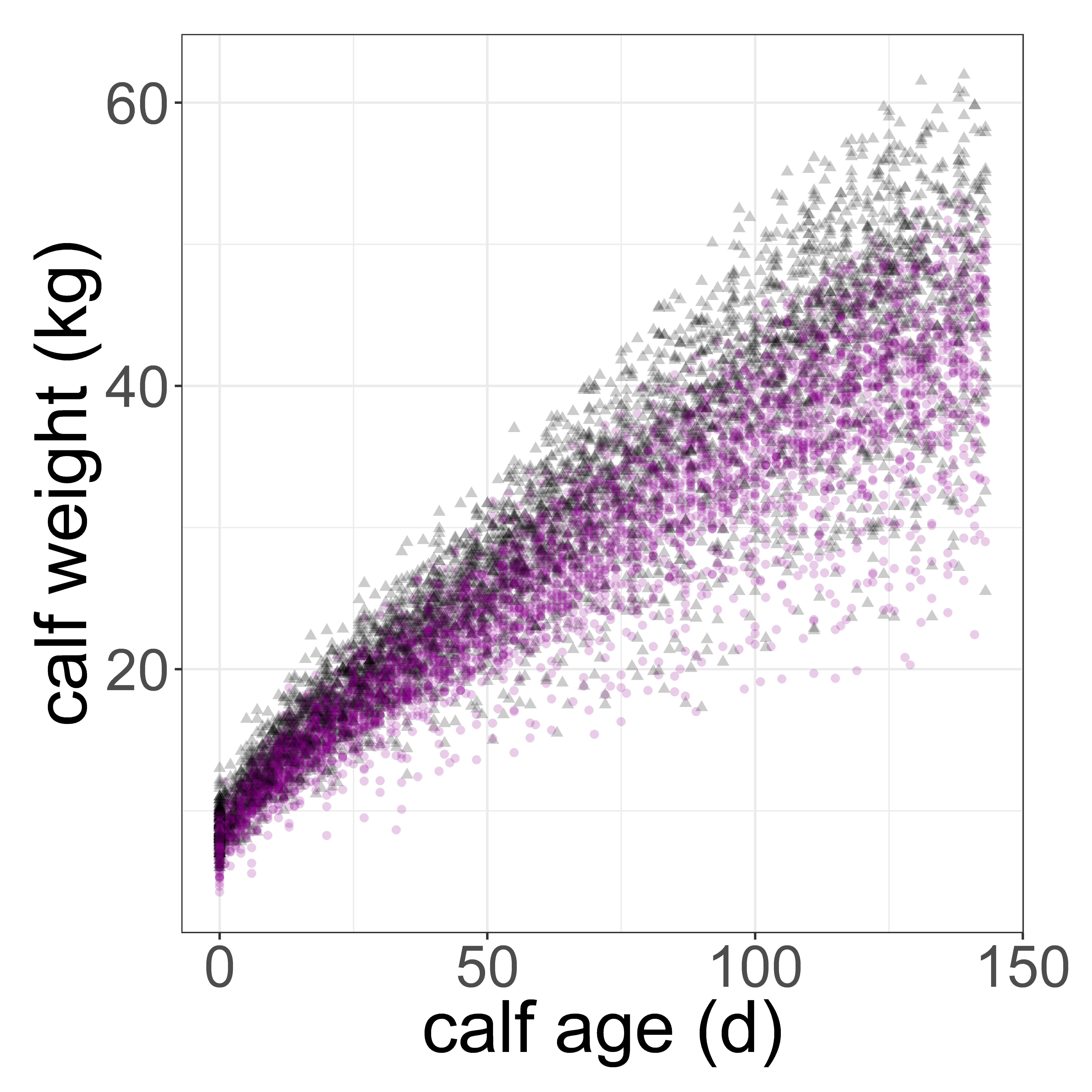

Supplement: S3 Fig — Actual calf weight (kg) versus calf age (d) across the first 143 days of life in males and females of Iberian red deer. Grey triangle: male calf; magenta circle: female calf. (JPEG) [file pone.0233809.s003.jpeg]

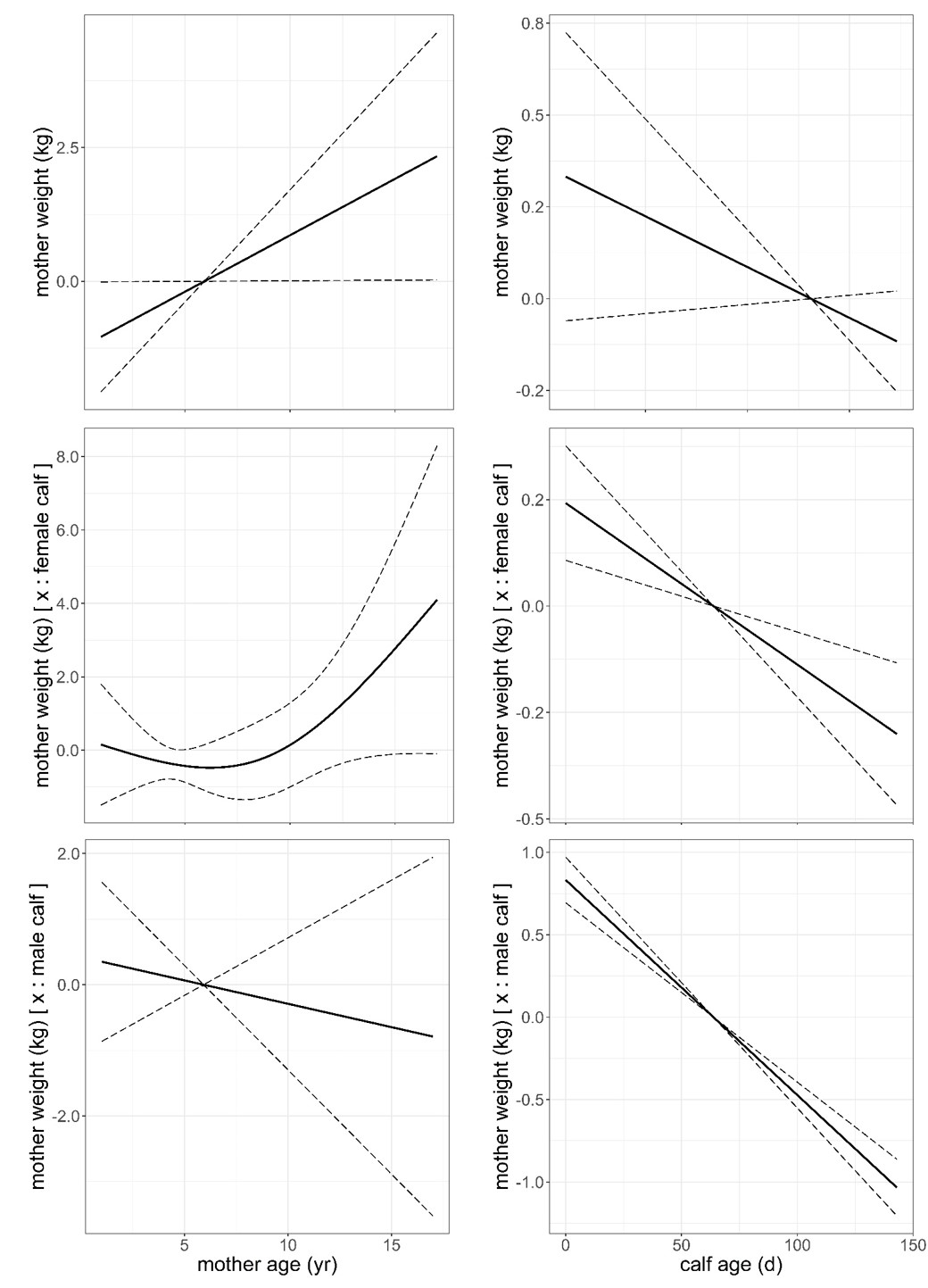

Supplement: S4 Fig — Response of mother body weight through lactation (from birth to forced weaning day 143) to mother age (years) and calf age (days) of the final model in Table 5, all other variables in the model fixed at their mean values. Dashed line: ± standard error. Response variable has been centred by subtraction of the mean value. (JPG) [file pone.0233809.s004.jpg]
